# Comparative effectiveness analysis of univariate time-series forecasting models for disease mortality rates in the global burden of disease database: a case study of global hypertensive heart disease among women of childbearing age

**DOI:** 10.3389/fpubh.2025.1681569

**Published:** 2025-11-27

**Authors:** Qi Deng, Songmei Wang, Jing Lyu

**Affiliations:** 1 Department of Physiology, School of Basic Medicine, Kunming Medical University, Kunming, Yunnan, China; 2 School of Public Health, Kunming Medical University, Kunming, Yunnan, China

**Keywords:** disease burden, forecasting, ARIMA, Prophet, LSTM, XGBoost

## Abstract

**Objective:**

The mortality rate of hypertensive heart disease (HHD) among women of childbearing age (WCBA) worldwide is continuously increasing. Accurate prediction of the mortality rate of HHD among WCBA globally plays a crucial role in evaluating the effectiveness of intervention measures and predicting future disease trends. To date, there has been few systematic comparative evaluations of prediction methods for epidemiological indicators in the field of disease burden. The purpose of this study was to systematically compare the performance of univariate prediction models in the global burden of disease (GBD) database.

**Method:**

Global mortality data on HHD in WCBA (1990–2021) were split into training and validation sets. We implemented and compared four models: AutoRegressive Integrated Moving Average (ARIMA), Prophet, eXtreme Gradient Boosting (XGBoost), and Long Short-Term Memory (LSTM). Model performance was assessed using Mean Squared Error (MSE), Mean Absolute Error (MAE), Mean Absolute Percentage Error (MAPE), and the Diebold-Mariano (DM) test for statistical significance.

**Results:**

The LSTM model demonstrated superior predictive accuracy on the validation set, with the lowest error rates across all metrics (MSE: 0.00021; MAE: 0.00872; MAPE: 0.662%). All the other models demonstrated statistically significant superiority over ARIMA (MSE: 0.03645; DM test *p* < 0.05 for all metrics). According to the DM test, both Prophet and LSTM demonstrated high predictive accuracy (*p* = 0.8762 for DM test based on MSE; *p* = 0.4292 for DM test based on MAE; *p* = 0.4303 for DM test based on MAPE). The LSTM model predicted that the mortality rate will exhibit an initial decline followed by a stabilization trend from 2022 to 2030, while the Prophet model predicted that the mortality rate will continue to rise.

**Conclusion:**

This study provided the first systematic comparison of univariate forecasting models for HHD mortality in WCBA using GBD data. A key finding was that both LSTM and Prophet performed exceptionally well statistically, LSTM achieves superior predictive capability via its gated mechanisms and state memory, while Prophet enhances interpretability through its additive model structure. This study therefore provides practical guidance for health authorities to select appropriate models based on actual needs to support improved resource planning for HHD.

## 1 Introduction

Global Burden of Disease (GBD) is a global resource created by WHO and the Institute for Health Metrics and Evaluation (IHME) to quantify health losses caused by hundreds of diseases, injuries, and risk factors (1). Hypertensive Heart Disease (HHD), which is a key component of cardiovascular disease burden of the GBD studies, often leads to characteristic structural and functional cardiac abnormalities, due to prolonged hypertension. The pathological changes of HHD includes left ventricular hypertrophy, myocardial fibrosis, impaired ventricular diastolic function, and heart failure (2). Women have a higher risk of hypertension than men, and their blood pressure regulation is more susceptible to estrogen fluctuations, menstrual cycle changes, and pregnancy-related hemodynamic changes than men (3, 4). The childbearing years represent a physiologically dynamic phase of female reproductive function, characterized by unique biological events including pregnancy, lactation, and potential adverse pregnancy outcomes. This life stage constitutes a high-risk period for the development of hypertension, with the elevated blood pressure subsequently increasing the susceptibility to HHD.

In 2015, the United Nations proposed Sustainable Development Goal 3, outlining the goal of reducing the global maternal mortality rate to below 70/100,000 by 2030 (5). Accurately predicting the future burden of HHD in women of childbearing age (WCBA) is crucial for guiding effective resource allocation and achieving relevant health goals. The value of predictive research has been demonstrated in studies at the regional level. For example, research by Qureshi et al., focus on cardiovascular disease mortality in Sindh, Pakistan, highlighted the critical importance of accurate predictions for quantifying the future disease burden of cardiovascular diseases, as well as for formulating health policies and allocating economic resources (6).

Optimizing forecasting methods is crucial for improving prediction accuracy. Relevant research has been widely conducted in many fields. Multilayer Perceptrons (MLP) have shown better performance than traditional models in the economic and financial forecasting (7, 8). Ensemble learning, hybrid models, and deep neural networks have demonstrated higher accuracy in environmental, fintech, and macroeconomic projection (9–12). Several medical studies have shown that machine learning and hybrid models are more accurate than traditional methods in the projection for infectious disease (13–15). Machine learning plays a more and more important role in modern forecasting, especially when handling complex time-series forecasting tasks.

Although the methodological optimization based on machine learning models has demonstrated significant potential in the field of public health, the systematic methodological research for the prediction based on GBD remains to be improved. The prior research has predominantly focused on conventional time series approaches-particularly AutoRegressive Integrated Moving Average (ARIMA), age-period-cohort (APC) models, and their Bayesian derivatives. These traditional methodologies failed to capture nonlinear relationships and multifactorial interactions adequately to represent the inherent complexity of disease burden dynamics (16), especially when analyzing disease burden trends that exhibit nonlinear patterns or trend reversals. Li Wang and Dan Liang et al. used variables such as age, gender, year, population size and related risk factors to train the XGBoost model, combining with using Shapley Additive Explanations (SHAP) to decompose the contribution of each variable to the disease, in the studies of iodine deficiency, iron deficiency and diarrhea (17–19). Although these studies expand the selection range of prediction model for GBD, they were lack of benchmark model comparisons to determine whether the proposed improvements actually enhanced the predictive performance. Jinyi Wu et al. compared the performance of different models in the study on the disease burden of femoral fractures based on GBD (20), which was lack of the description of the training set and verification set of the model. And the selection of the time window during model training was also absent. Moreover, the input variables are vague and unrestricted. These problems may lead to bias in the evaluation of models.

Our study carried out a multi-model comparison experiment under the framework of univariate model for the first time. The analysis was rigorously confined to using mortality rates of HHD from WCBA (1990–2021) as the sole input variables, thereby mitigating potential confounding factors from other covariates. The dataset was split into training (1990–2015) and validation (2016–2021) sets. Model performance was then assessed by comparing predicted values with ground truth.

The model selection was based on the following rationale: ARIMA: A classical time series forecasting model in disease burden research, capable of capturing linear trends and serving as the baseline model in this study. Prophet: Analysis of long-term trends and periodicity through an additive regression model, with built-in support for seasonality and changepoint detection, to evaluate structured time series models (21). XGBoost: A powerful tree-based ensemble algorithm that has proven particularly effective for time series forecasting tasks (22). There have been few studies applying XGBoost with univariate input in disease burden research before. Our study tested its applicability to univariate time series. LSTM: A recurrent neural network architecture designed to capture nonlinear temporal dynamics to evaluate the generalization capability of deep learning models on univariate time series (23).

Our study employed a controlled-variable design to systematically compare the inherent performance differences among machine learning, traditional statistical methods, and structured temporal models, for disease burden forecasting using identical univariate inputs. This approach addressed a critical methodological gap in the field by providing robust empirical evidence for model selection in temporal health data analytics. The prediction findings of HHD in the vulnerable population, WCBA, of this study inform targeted global health interventions and help policymakers optimize healthcare resource allocation strategies.

## 2 Methods

Statistical models (ARIMA), Machine learning models (XGBoost, LSTM), and the Prophet forecasting procedure were used for modeling and the prediction of the mortality rate of HHD among WCBA.

### 2.1 Data sources

Mortality data of females aged from 15 to 49 years of GBD database^1^ were used for the analysis of the global burden of HHD among WCBA. Univariate modeling typically refers to prediction models that rely solely on the time series itself without incorporating external covariates. The univariate modeling was used for the prediction of the global mortality rate of HHD among WCBA, where future values $y_{t},y_{t+1},\ldots ,y_{t+d}$ were forecasted based solely on historical temporal patterns $y_{1},y_{2},\ldots ,y_{t-1}$ , with d denoting the prediction window length (in days).

### 2.2 Data splitting

The global mortality rate time series of HHD among WCBA from 1990 to 2021 was partitioned into the training set and the validation set. The data from 1990 to 2015 was used for model training and parameter estimation. And the data from 2016 to 2021, constituting approximately 30% of the total series, were selected as a validation set. This temporally separated validation set was crucial for providing an unbiased evaluation of the models’ predictive performance on unseen, external data and for mitigating the risk of overfitting.

### 2.3 Forecasting models

Four different models were employed for modeling, followed by comparative effectiveness analysis. The four models with their respective modeling platforms were: ARIMA (R 4.4.3), Prophet (Python 3.1.1), XGBoost (Python 3.1.1), and LSTM (Python 3.1.1).

The detailed descriptions of each model were as follows:

#### 2.3.1 ARIMA

As a classical time-series forecasting approach, ARIMA is particularly suitable for stationary time-series data or data sequence that can be made stationary through differencing. Given its extensive use as a traditional statistical model within the GBD database system, ARIMA was selected as the benchmark model here to represent how conventional statistical modeling methods perform in prediction.

The ARIMA model can be expressed as:

$$\begin{array}{l} y_{t}=c+\varphi_{1}y_{t-1}+\varphi_{2}y_{t-2}+\cdots +\varphi_{p}y_{t-p}+a_{t}\\ -\theta_{1}a_{t-1}-\theta_{2}a_{t-2}-\cdots -\theta_{q}a_{t-q} \end{array}$$

where c is a constant, $y_{t}$ is the observed value of the time series and p represents the order of the auto-regressive (AR). $\varphi_{1},\varphi_{2},\cdots ,\varphi_{p}$ are the coefficients of the AR. q is the order of the moving average (MA). $\theta_{1},\theta_{2},\cdots ,\theta_{q}$ are the coefficients of the MA. A fundamental assumption of the model is that the random error term $a_{t}$ follows a white noise process, being independently and identically distributed with a mean of zero and a constant variance $\sigma^{2}$ .

R 4.4.3 was used for the analysis with visual inspection of the time series to identify trends, followed by formal stationarity testing using Augmented Dickey-Fuller (ADF) tests. Autocorrelation (ACF) and partial autocorrelation (PACF) plots were then checked for guiding initial parameter selection and detecting the underlying structure of the time series.

Given that the auto.arima function represents a commonly employed and objective approach for ARIMA modeling in studies utilizing the GBD database, it was applied in the study to ensure the ARIMA model was directly comparable to common practice and to minimize subjective bias in model specification.

Model quality was assessed using the Akaike Information Criterion (AIC) before using the auto.arima function from the forecast package to automatically determine the optimal ARIMA specification, which produced the ARIMA (0,1,1) model. And the final validation was confirmed through residual diagnostics including autocorrelation analysis and Ljung-Box testing. All of these supported the adequacy of the model, as the residuals exhibited characteristics of white noise without significant autocorrelation patterns.

#### 2.3.2 Prophet

Prophet is a structured additive time series model that explicitly decomposes data into interpretable components (trend, seasonality, and anomalies), integrating strengths of classical statistical methods and machine learning algorithms. As global HHD mortality data for WCBA exhibits non-stationary characteristics due to the influence of policy changes and advancements in healthcare, this study adopts Prophet to alleviate the effects of anomalous data points. Furthermore, this model was employed to investigate the efficacy of structured time series modeling for univariate historical data.

The mathematical formulation of the Prophet model follows an additive decomposition framework, expressed as:

$$y(t)=g(t)+s(t)+h(t)+\varepsilon_{t}$$

where y(t) represents the observed value at time t . g(t) denotes the trend component modeling long-term growth. s(t) captures seasonal variations (daily, weekly, yearly). h(t) accounts for holiday and special event effects. $\varepsilon_{t}\;$ is the error term assumed to be normally distributed.

The trend component is mathematically formulated as:

$$g(t)=(k+u{\left(t\right)}^{\mathrm{T\delta}})t+(\mu +u{\left(t\right)}^{\mathrm{T\gamma}})$$

where k is the growth rate, μ is the offset parameter and u(t) is a vector of adjustment factors at time t to account for changepoints. δ and γ are vectors of parameters that model the rate and offset adjustments, respectively.

The seasonal component is mathematically formulated as:

$$s(t)=\sum_{n=1}^{N}(c_{n}\cos(\frac{2\mathrm{\pi nt}}{P})+d_{n}\sin(\frac{2\mathrm{\pi nt}}{P}))$$

where N is the number of Fourier terms used to approximate the seasonality (higher N allows for more complex seasonal patterns). $c_{n}$ and $d_{n}$ are the Fourier coefficients for the n -th term, which are learned from the data. P is the period of the seasonal component.

Using Prophet in Python 3.1.1, we formatted the data with years as “ds” (datetime) and mortality rates as “y” per package requirements. The model was initialized with yearly_seasonality = True to account for annual patterns, while disabling weekly and daily seasonality given the yearly timestamp resolution.

#### 2.3.3 XGBoost

Both L1 (lasso) and L2 (ridge) regularization terms are integrated into XGBoost, enabling it to effectively alleviate overfitting risks when modeling HHD mortality data for WCBA. This regularization framework enhances the model’s generalizability for future mortality predictions while improving forecasting accuracy and stability. As a state-of-the-art traditional machine learning algorithm, XGBoost was employed in this study to evaluate the performance of conventional machine learning approaches in modeling univariate historical time series data.

The mathematical formulation of XGBoost is as follows:

$$\hat{y_{i}}=\sum_{j=1}^{K}(f_{j})(X_{i})$$

where $\hat{y_{i}}$ is the predicted value for the i -th sample. $y_{i}$ is the corresponding true observed value. K is the number of decision trees, and $f_{j}(X_{i})$ represents the predicted value from the j -th decision tree for the i -th sample.

The model was implemented using the XGBoost library in Python 3.11, configured for regression tasks (objective = “reg:squarederror”) with mean squared error as the loss function. To enable multi-step forecasting (9-year horizon), we employed scikit-learn’s MultiOutputRegressor wrapper, which trained separate XGBoost models for each output step while maintaining temporal dependencies. The XGBoost model was initialized with 100 decision trees (n_estimators = 100) to control ensemble size. Default hyperparameters included: a learning rate of 0.01 to moderate the contribution of each tree, maximum tree depth (max_depth) of 3 to limit model complexity, and L1/L2 regularization terms ( α =0, λ =1) to balance between model flexibility and generalization capability. These parameter settings were selected through empirical, iterative manual tuning to optimize predictive performance while preventing overfitting. The model was trained using varying time windows (i.e., different historical data lengths) to predict future outcomes, while maintaining a fixed forecast horizon consistent with the validation set. This approach ensured direct comparability between training and validation results.

#### 2.3.4 LSTM

The LSTM network’s gated architecture—featuring forget gates, input gates, and output gates-provides unique capabilities for capturing long-range temporal dependencies. This structure makes LSTMs particularly robust when handling noisy real-world datasets and modeling complex nonlinear relationships in time series data. Given the high noise levels and complex nonlinear relationships inherent in our dataset, this study employs the LSTM network for predictive modeling. As a canonical deep learning framework, LSTM is especially suitable for assessing the effectiveness of deep neural networks in the modeling process of univariate historical time-series data. The LSTM mechanism comprises three fundamental components: the forget gate, the input gate, and the cell state, with its mathematical formulation expressed as:

forget gate:

$$f_{t}=\sigma (W_{f}\cdot [h_{t-1},x_{t}]+b_{f})$$

where $f_{t}$ represents the forget gate’s output at time step *t*, σ denotes the activation function, $W_{f}$ is the weight matrix of the forget gate, $h_{t-1}$ indicates the model’s output at time step *t-1*, $x_{t}$ corresponds to the input at time step t, and $b_{f}$ signifies the bias term of the forget gate.

input gate:

$$i_{t}=\sigma (W_{i}\cdot [h_{t-1},x_{t}]+b_{i})$$

where $i_{t}$ represents the input gate’s output at time step t, $W_{i}$ denotes the input gate’s weight matrix, and $b_{i}$ corresponds to the input gate’s bias term.

candidate cell state:

$${\tilde{C}}_{t}=\tanh(W_{C}\cdot [h_{t-1},x_{t}]+b_{C})$$

where ${\tilde{C}}_{t}$ represents the candidate cell state at time step *t*, tanh denotes the hyperbolic tangent activation function, $W_{C}$ corresponds to the weight matrix for the candidate state computation, and $b_{C}$ signifies the bias term of the candidate cell state.

updated cell state:

$$C_{t}=f_{t}\cdot C_{t-1}+i_{t}\cdot {\tilde{C}}_{t}$$

where $C_{t}$ denotes the cell state at time step t, and $C_{t-1}$ represents the cell state at time step t−1 .

output gate:

$$o_{t}=\sigma (W_{o}\cdot [h_{t-1},x_{t}]+b_{o})$$

where $o_{t}$ denotes the output at time step t, $W_{o}$ represents the weight matrix of the output gate, and $b_{o}$ corresponds to the bias term of the output gate.

Hidden state (i.e., output):

$$h_{t}=o_{t}\cdot \tanh(C_{t})$$

The LSTM model was implemented in PyTorch to process univariate mortality rate time series (input_size = 1) through a two-layer architecture (num_layers = 2) with 64 hidden units per layer (hidden_size = 64), configured in batch-first format (batch_first = True). The network’s final output was projected to 6 dimensions via a fully-connected layer (output_size = 6) to generate annual mortality predictions for 2016–2021. The same empirical tuning approach as for XGBoost was applied. Model training employed mean squared error (MSE) loss minimization using the Adam optimizer (learning_rate = 0.001), with temporal validation performed through sliding-window training on 1990–2015 data and fixed-horizon evaluation on 2016–2021 observations.

### 2.4 Model evaluation

#### 2.4.1 Evaluation metrics

To systematically evaluate the predictive performance of the models, this study employed three metrics for comprehensive comparison: MSE, Mean Absolute Error (MAE), and Mean Absolute Percentage Error (MAPE). All the three metrics are inversely correlated with prediction accuracy, meaning lower values indicate better model performance. The evaluation indicators are presented in Table 1.

**Table 1:** Univariate forecasting error metrics for global HHD mortality among WCBA.

No	Error	Equations
i	MAE	MAE=1n∑l=1n∣el−el^∣
ii	MSE	$\mathrm{MSE}=\frac{1}{n}\sum_{l=1}^{n}{\left(e_{l}-\hat{e_{l}}\right)}^{2}$
iii	MAPE	$\text{MAPE}=\frac{100\%}{n}\sum_{l=1}^{n}|\frac{e_{l}-\hat{e_{l}}}{e_{l}}|$

Where n is the total number of observations, $e_{l}$ is the actual observed value for the l -th observation, and $\hat{e_{l}}$ is the predicted value for the l -th observation.

The MAPE was expressed as a percentage and served as the primary standard for accuracy classification. MAPE<10% indicated high prediction accuracy, 10% < MAPE<20% indicated good accuracy, 20% < MAPE<50% indicated reasonable accuracy, MAPE≥ 50% indicated poor accuracy (24). MAPE<20% was set as the threshold for determining satisfactory prediction accuracy, based on these established criteria and the specific accuracy requirements of the study.

#### 2.4.2 Statistical significance test

To determine whether the differences in forecast accuracy between the models are statistically significant, the Diebold-Mariano (DM) test was employed under MSE, MAE and MAPE loss functions (16). The DM statistic is calculated as follows:

$$\mathrm{DM}=\frac{\bar{D}}{\sqrt{\left(S^{2}/n\right)}}$$

where $\bar{D}=\frac{1}{n}\sum_{t=1}^{n}D_{t}$ is the sample mean of the loss differential series. $D_{t}=L({\varsigma_{t}}^{\left(1\right)})-L({\varsigma_{t}}^{\left(2\right)})$ denotes the loss differential at time t . Here, L(.) is the loss function, and ${\varsigma_{t}}^{\left(1\right)}$ and ${\varsigma_{t}}^{\left(2\right)}$ are the forecast errors of the two models compared. $S^{2}$ is the estimated long-run variance of the differential series $\left\{D_{t}\right\}$ . This estimation is robust to autocorrelation and heteroscedasticity, often computed using the Newey-West heteroscedasticity- and autocorrelation-consistent (HAC) estimator. n is the number of forecast observations.

The hypothesis for the DM test is:

*H₀*: The two models being compared are equally accurate.

*H₁*: The model in the column is more accurate than the model in the row.

The test was conducted pairwise for all model combinations at a significance level of *α* = 0.05. A *p*-value less than 0.05 leads to the rejection of the null hypothesis, indicating a statistically significant difference in forecasting performance.

### 2.5 Further forecasting and visualization

Four types of models (ARIMA, Prophet, XGBoost and LSTM) were used for the projection of disease burden of HHD among WCBA from 2022 to 2030. Regarding visualization, all figures were generated using R 4.4.3, except for the Prophet prediction plot which was created with Python 3.3.1.

## 3 Results

### 3.1 Validation results of the ARIMA, prophet, XGBoost, and LSTM models on global HHD mortality data among WCBA during 2016–2021

#### 3.1.1 Validation results of the ARIMA and prophet models

**Figure 1:**
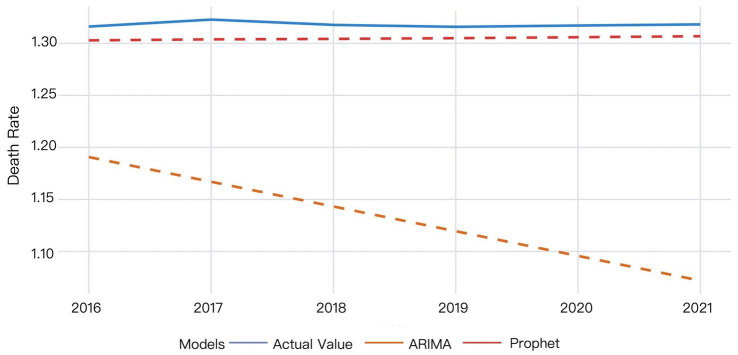
The validation results of the ARIMA and Prophet models on 2016–2021 data.

The validation results are presented in Figure 1.

The ARIMA model exhibited larger deviations between predicted and actual values that progressively widened over time, whereas the Prophet model demonstrated superior predictive accuracy. According to the Prophet model, the actual mortality rate in 2016 was slightly higher than the predicted value. The actual mortality rate then showed a minor increase, reaching the peak value in 2017, while the predicted values remained stable, indicating the model’s partial failure to capture this temporal variation. The actual mortality rate then decreased and gradually became relatively stabilized since 2018, while the predicted values showed a slow upward trend, yet consistently underestimated the true values. The difference between the actual and predicted mortality rates decreased by 2021, though the predictions remained below actual measurements (Figure 1).

#### 3.1.2 XGBoost model validation results

**Figure 2:**
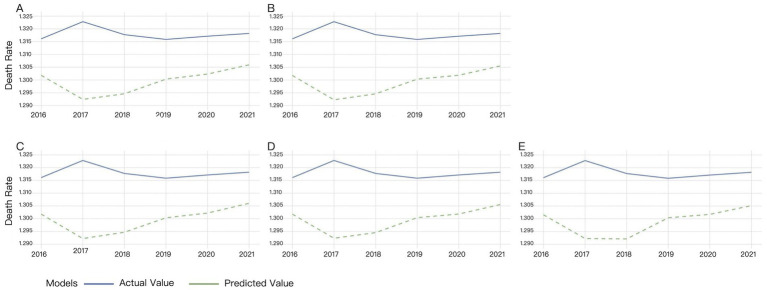
Validation results of XGBoost under different time windows. **(A)** 4-day window, **(B)** 6-day window, **(C)** 8-day window, **(D)** 10-day window, **(E)** 12-day window.

Multiple time windows (4, 6, 8, 10, and 12 days) were adopted for XGBoost model validation, with the visual results presented in Figure 2.

Different time windows had minimal impact on the XGBost model. Figure 2 showed that XGBoost demonstrated low sensitivity to time window size variations. The discrepancy between actual and predicted mortality rates progressively widened from 2016 to 2017, while it gradually narrowed since 2018. The predicted mortality trend largely aligned with actual observations from 2019 to 2021, though the predicted values consistently remained below the true measurements (Figure 2).

#### 3.1.3 LSTM model validation results

**Figure 3:**
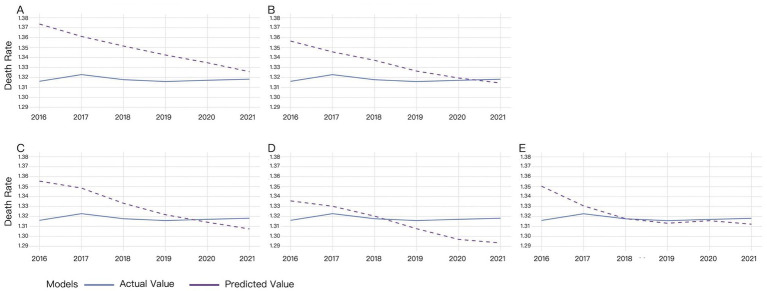
Validation results of LSTM under different time windows. **(A)** 4-day window, **(B)** 6-day window, **(C)** 8-day window, **(D)** 10-day window, **(E)** 12-day window.

Multiple time windows (4, 6, 8, 10, and 12 days) were adopted for LSTM model validation, with the comparative results visualized in Figure 3.

For the 4-day time window, there was a significant difference between the actual and predicted from 2016 to 2017. The difference gradually decreased since 2018, reaching its minimum in 2021. The predicted values remained higher than the actual values (Figure 3A).

For the 6-day time window, predicted mortality rates consistently overestimated actual values from 2016 to 2020, though the discrepancy gradually diminished, reaching optimal alignment in 2020. A reversal occurred with predicted rates underestimating actual mortality, demonstrating divergent trends in 2021 (Figure 3B).

For the 8-day time window, predicted mortality rates systematically overestimated actual values from 2016 to 2019, with the magnitude of overestimation decreasing annually. A crossover occurred in 2020, where predictions shifted to underestimation, exhibiting an inverse trend relative to actual values (Figure 3C).

For the 10-day time window, predicted mortality rates exhibited slightly consistent overestimation relative to actual values from 2016 to 2018. A reversal occurred since 2019, with predictions underestimating actual mortality rates and the divergence progressively widening by 2021 (Figure 3D).

For the 12-day time window, the predicted mortality rate was higher than the actual mortality rate from 2016 to 2018, while the difference between the predicted and actual values gradually decreased. The predicted value was almost the same with the actual value in 2018. Then the difference between the predicted and actual values slightly increased, with the predicted values lower than the actual values (Figure 3E).

The results demonstrate that the LSTM model’s predictive accuracy improves with increasing time window sizes, achieving near-perfect alignment with ground truth values under the 12-day window configuration. Only marginal deviations were observed in 2016 and 2021 (Figure 3).

**Figure 4:**
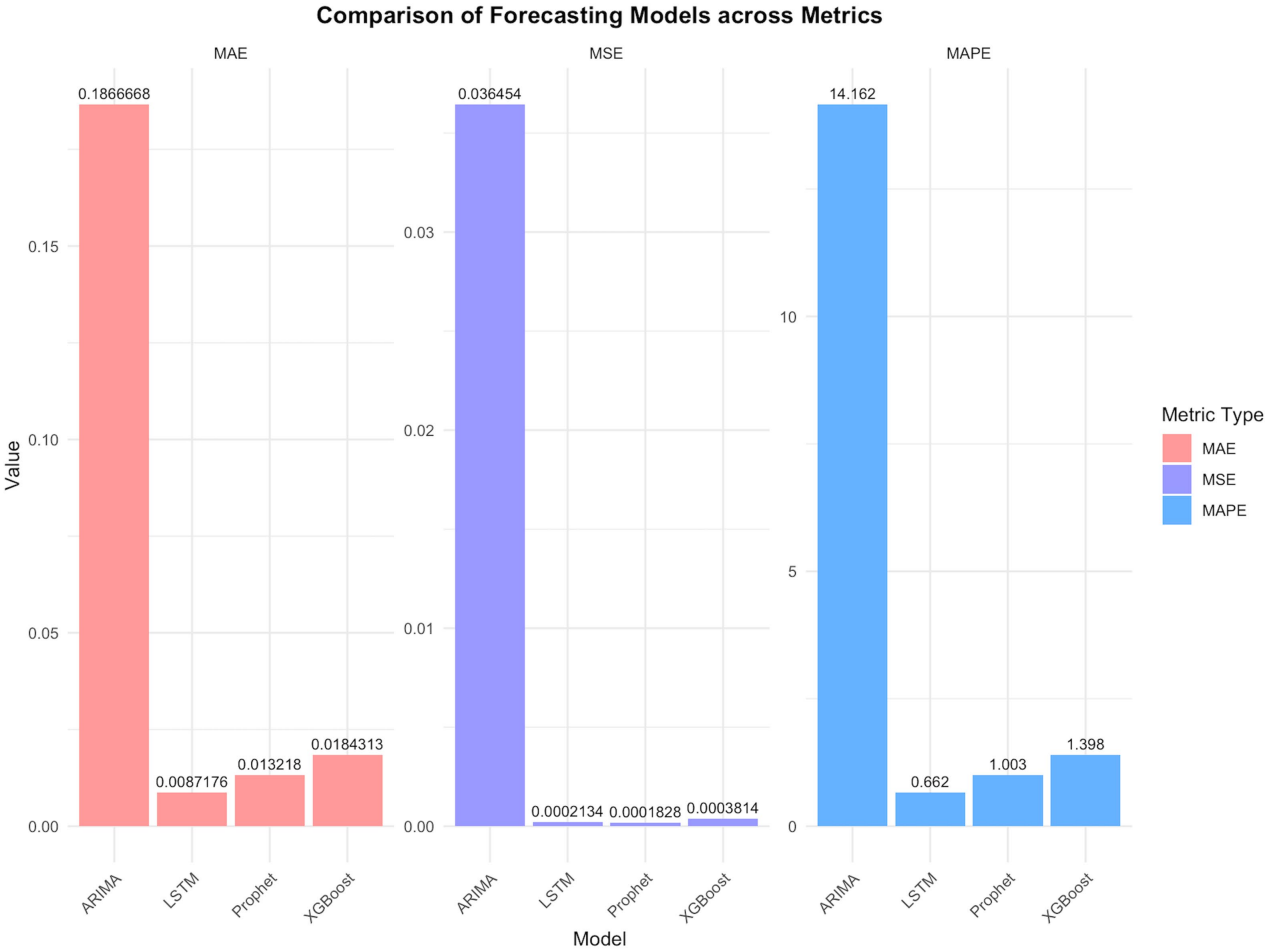
Comparisons of models using key performance indicators.

Validation results based on MSE, MAE, and MAPE metrics under optimal time window conditions are presented in Table 2 and Figure 4.

**Table 2:** Comparison of model forecasting accuracy for HHD mortality in WCBA on the validation set (2016–2021).

Model/evaluation metrics	MSE	MAE	MAPE (%)
ARIMA	0.03645	0.18667	14.162
Prophet	0.00018	0.01322	1.003
XGBoost	0.00038	0.01843	1.398
LSTM	0.00021	0.00872	0.662

The DM test provided the statistical evidence for the difference in the performance of the four types of models in the error metrics (Tables 3–5).

**Table 3:** *P*-values of the DM test for pairwise model comparisons (using MSE as the loss function).

Models	ARIMA	Prophet	XGBoost	LSTM
ARIMA	0	0.0030	0.0032	0.0032
Prophet	0.0030	0	0.0762	0.8762
XGBoost	0.0032	0.0762	0	0.5441
LSTM	0.0032	0.8762	0.5441	0

**Table 4:** *P*-values of the DM test for pairwise model comparisons (using MAE as the loss function).

Models	ARIMA	Prophet	XGBoost	LSTM
ARIMA	0	0.0002	0.0003	0.0005
Prophet	0.0002	0	0.0341	0.4292
XGBoost	0.0003	0.0341	0	0.1946
LSTM	0.0005	0.4292	0.1946	0

**Table 5:** *P*-values of the DM test for pairwise model comparisons (using MAPE as the loss function).

Models	ARIMA	Prophet	XGBoost	LSTM
ARIMA	0	0.0002	0.0003	0.0005
Prophet	0.0002	0	0.0339	0.4303
XGBoost	0.0003	0.0339	0	0.1951
LSTM	0.0005	0.4303	0.1951	0

**Figure 5:**
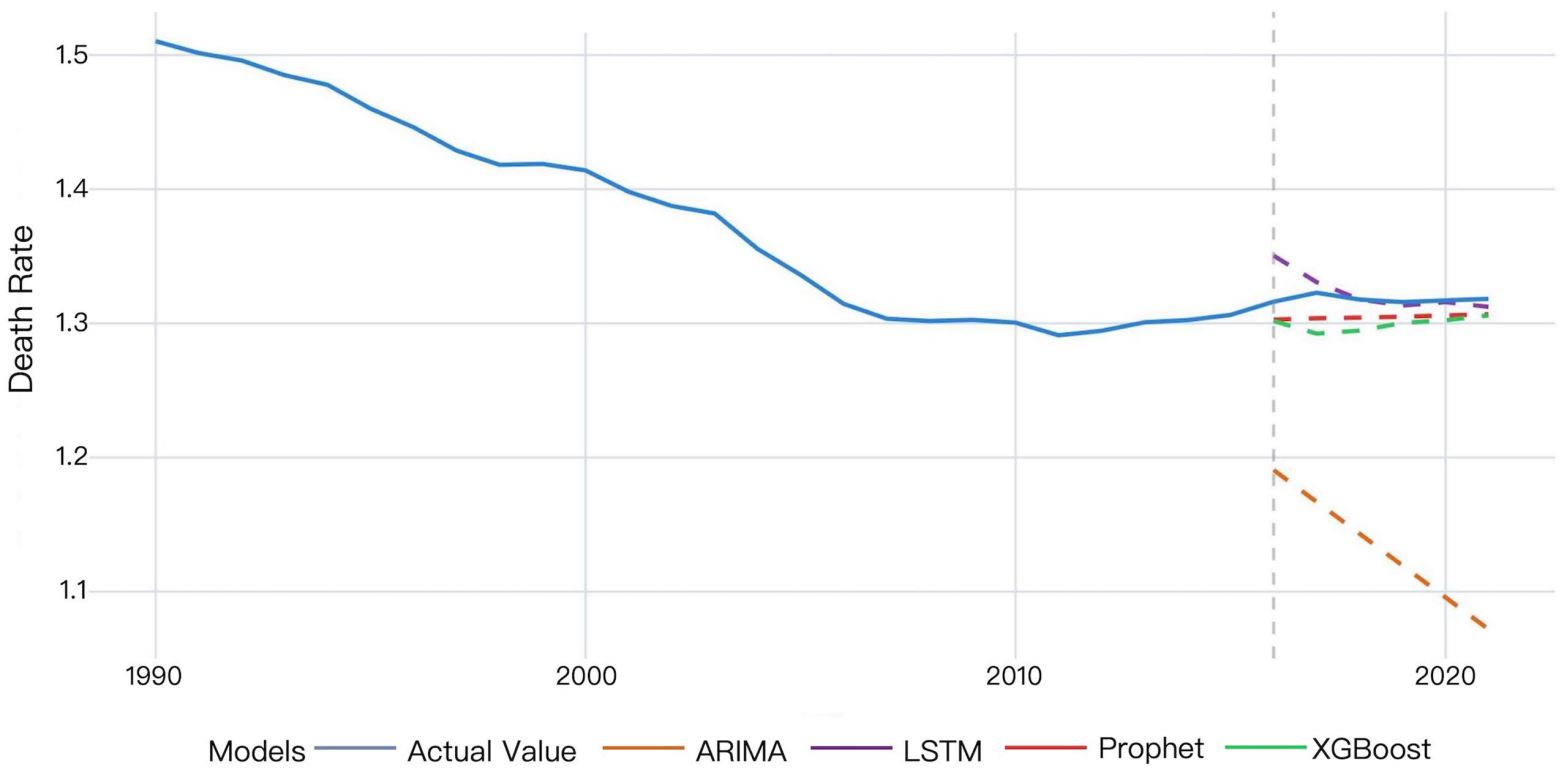
Comparative validation results of the four models for global HHD mortality among WCBA.

Validation results of the four models for global HHD mortality rates among WCBA during 2016–2021 are shown in Figure 5.

The MAPE values of four models were all below 20%, demonstrating prediction efficiency exceeding 80%. The LSTM model demonstrated superior predictive accuracy, with the lowest error rates across all metrics (MSE: 0.00021; MAE: 0.00872; MAPE: 0.662%), confirmed by the comparative visualization of MAE and MSE metric (Table 2 and Figures 4, 5). According to the DM test, both Prophet and LSTM demonstrated higher predictive accuracy than the other models (Tables 3–5). And there was no significant difference between Prophet and LSTM (*p* = 0.8762 for DM test based on MSE; *p* = 0.4292 for DM test based on MAE; and *p* = 0.4303 for MAPE). The prediction performance of XGBoost was significantly better than ARIMA (*p* = 0.0003 for DM test based on MAE), while weaker than Prophet (*p* = 0.0341 for DM test based on MAE and *p* = 0.0339 for DM test based on MAPE). And there was no significant difference between XGBoost and LSTM (*p* = 0.1946 for DM test based on MAE and *p* = 0.1951 for DM test based on MAPE). For ARIMA, the prediction performance was significantly weaker than the other models (MSE: 0.03645; DM test *p* < 0.05). In conclusions, Prophet and LSTM were the top-performing models. XGBoost was also a powerful prediction model better than the traditional methods. The model selection of Prophet and LSTM in application was suggested to be determined by the non-performance factors, such as interpretability and computational efficiency.

### 3.2 Predictive analysis of global HHD mortality among WCBA from 2022 to 2030 using ARIMA, prophet, XGBoost, and LSTM models

**Figure 6:**
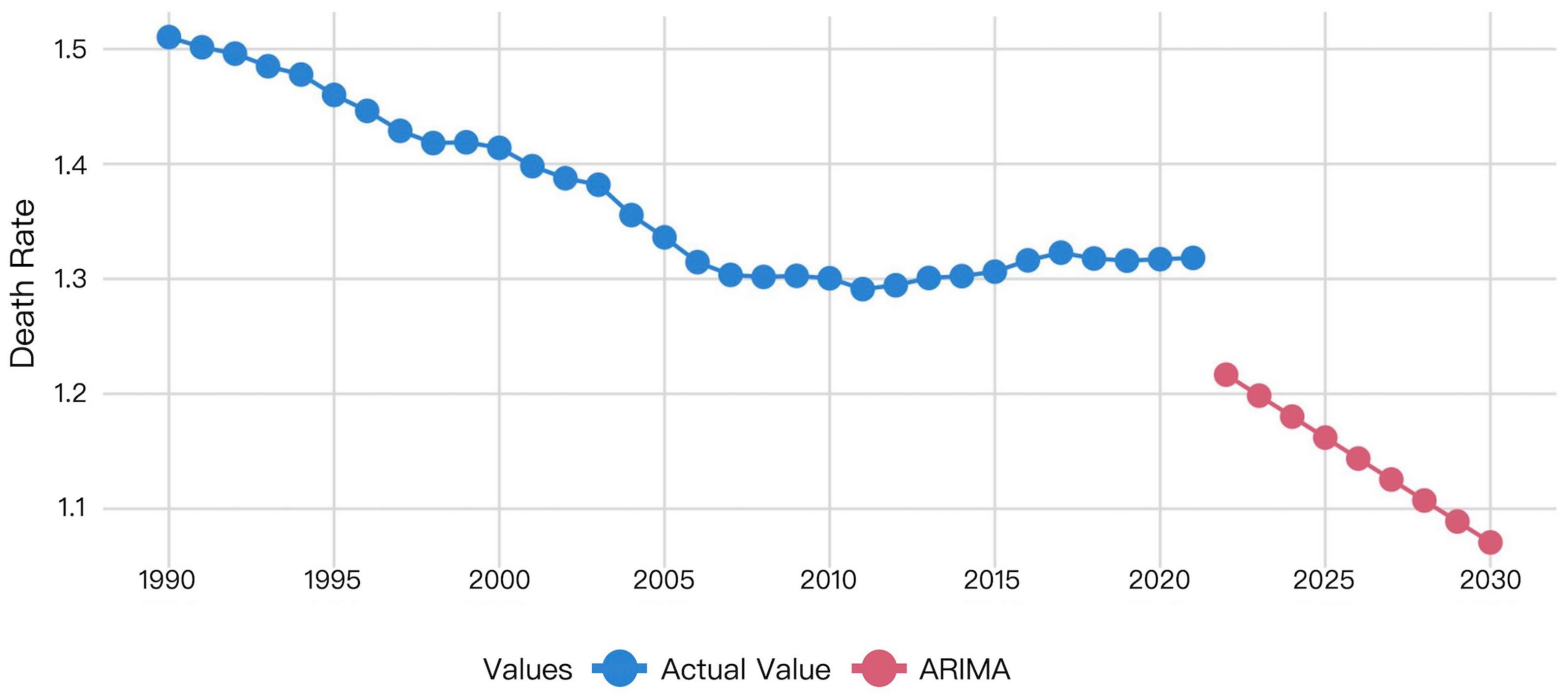
Visualization of ARIMA model prediction results.

**Figure 7:**
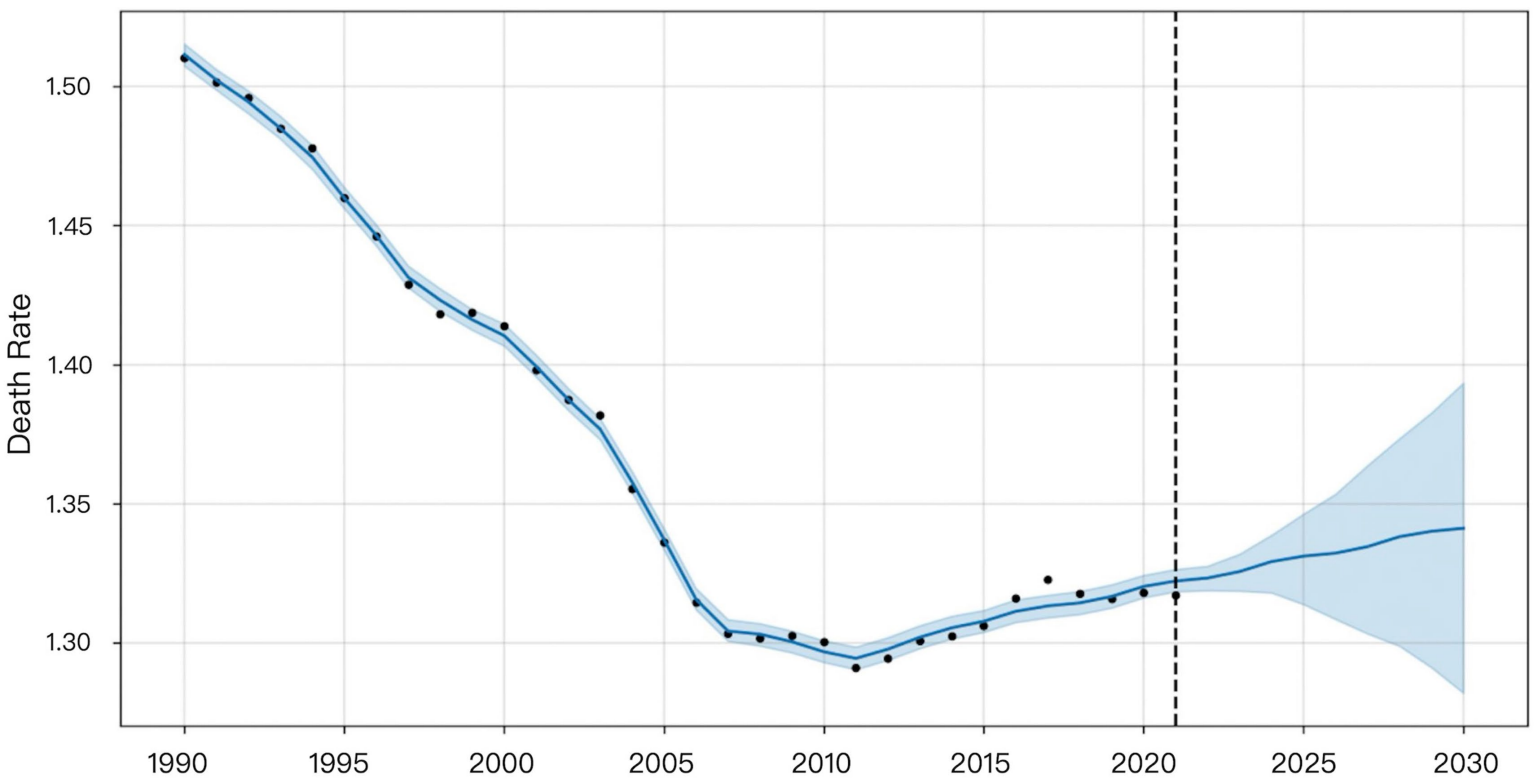
Visualization of Prophet model prediction results.

**Figure 8:**
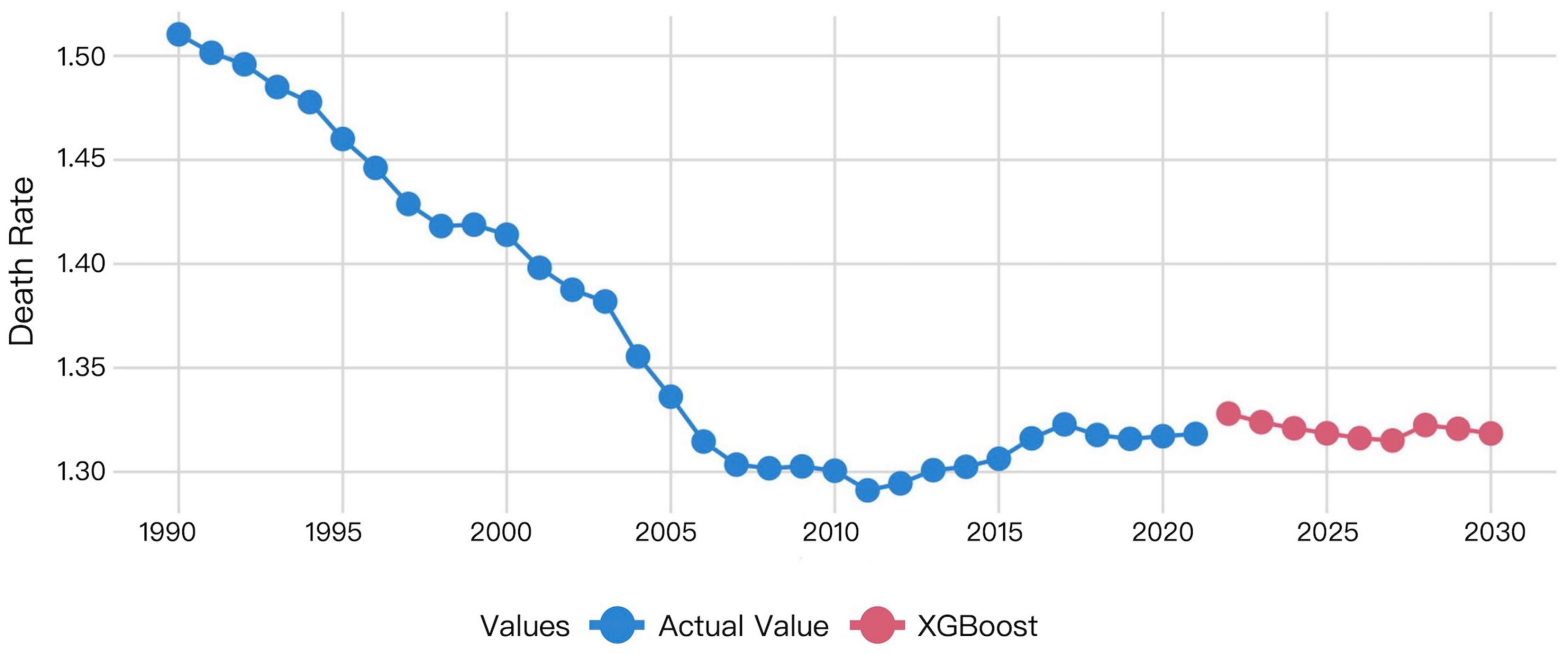
Visualization of XGBoost model prediction results.

**Figure 9:**
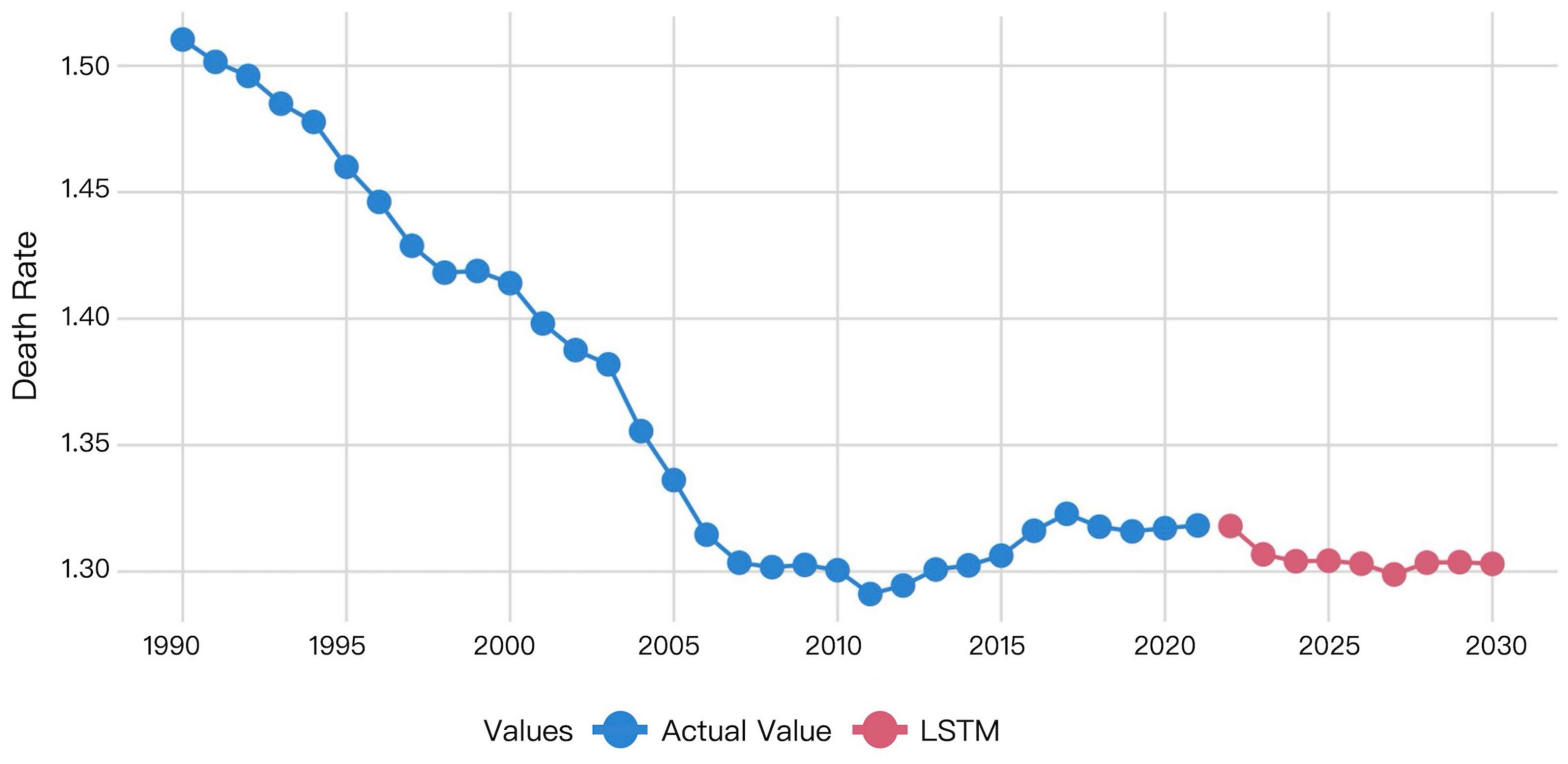
Visualization of LSTM model prediction results.

The XGBoost and LSTM models employed their respective optimal time windows identified during validation as the superior hyperparameters for predicting global HHD mortality among WCBA from 2022 to 2030. The comparative forecasting results of ARIMA, Prophet, XGBoost, and LSTM are shown in Figure 6–9.

Figure 6 indicated that the ARIMA model predicts a gradual decline in HHD mortality among WCBA from 2022 to 2030.

Figure 7 indicated that the Prophet model predicts a gradual increase in HHD mortality among WCBA from 2022 to 2030.

The XGBoost prediction results revealed a notable discrepancy between actual 2021 mortality rates and 2022 forecasts. This predicated mortality exhibited a progressive decline from 2022 to 2027, followed by a transient increase in 2028 before decrease (Figure 8).

The LSTM model predictions demonstrated that the predicted mortality rate in 2022 was closely aligned with the actual mortality rate in 2021. Subsequently, the predicted mortality exhibited a downward trend from 2022 to 2023, and remained essentially stable from 2024 to 2030, at levels consistent with the 2023 forecast (Figure 9).

Table 6 showed the predicted values for global HHD mortality among WCBA from 2022 to 2030. The ARIMA model projected a declining trend. The Prophet model forecasted an increasing trend. XGBoost predictions showed mortality decreasing from 2022 to 2027 followed by an increase before declining again in 2030. The LSTM model predicted a decrease from 2022 to 2023 with subsequent stabilization since 2023. The prediction of the LSTM model was considered to be most reliable, due to the superior performance of it demonstrated by the former validation results.

**Table 6:** Prediction of global HHD mortality in WCBA by different models (2022–2030).

Model type/year	2022	2023	2024	2025	2026	2027	2028	2029	2030
ARIMA	1.21667	1.19842	1.18017	1.16193	1.14368	1.12543	1.10719	1.08894	1.07069
Prophet	1.32339	1.32571	1.32929	1.33125	1.33233	1.33466	1.33823	1.34019	1.34127
XGBoost	1.32799	1.32388	1.32094	1.31853	1.31619	1.31508	1.32250	1.32069	1.31844
LSTM	1.31799	1.30677	1.30426	1.30406	1.30309	1.29882	1.30350	1.30370	1.30303

## 4 Discussion

### 4.1 Feasibility of construction of the univariate models for global HHD mortality data among WCBA based on GBD

Univariate time series modeling takes the historical sequence itself as input to predict future values, with the general mathematical formulation:

$$\hat{y_{t}}=v(\{y_{1},y_{2},\ldots ,y_{t-1}\};\vartheta )$$

where ϑ represents the model parameters and v represents the prediction model or function.

Time series data consists of sequentially recorded measurements collected at regular time intervals. The temporally ordered observations can be either a single measurement variable (univariate) or multiple interrelated variables (multivariate) (25). Panel data typically refer to observations collected for the same set of individuals or units across multiple time points, combining characteristics of both cross-sectional data and time-series data. Grouped data (also known as clustered or stratified data) involve partitioning the dataset into distinct groups (e.g., countries, regions, age cohorts), where observations within each group share similar features or structures. A Lancet study in 2015 by the GBD Collaboratives demonstrated that disease-specific mortality rates in the GBD database are stratified by time (year) and geographic region (country), conforming to a panel data structure while exhibiting characteristics of grouped data. These data represent small-sample, univariate time series (26). This study validates the feasibility of univariate modeling for small-sample time series data using global HHD mortality rates among WCBA (1990–2021). The DM test stratifies model performance into three distinct tiers: (1) Top Tier (Statistically Equivalent): LSTM and Prophet. (2) Middle Tier: XGBoost, which was significantly better than ARIMA but showed mixed results against the top tier. (3) Bottom Tier: ARIMA. The difference in performance demonstrates that modern flexible models, including structured time series and deep learning, are more suitable for the forecasting task than traditional statistical or tree-based models.

Notably, the statistical equivalence between LSTM and Prophet, as revealed by the DM test, can be further nuanced by the choice of loss function, with each metric highlighting different model strengths. The MSE heavily penalizes large deviations, making it sensitive to outliers. The MAE is more robust to extreme values. In contrast, the MAPE provides a scale-invariant comparison, which is particularly useful for understanding relative forecasting accuracy. The fact that LSTM and Prophet remain statistically equivalent across these diverse metrics—MSE, MAE, and MAPE—suggests that their performance parity is not an artifact of a single evaluation perspective but is robust to different measurements of error. This underscores that both models capture the underlying data generating process effectively, albeit through different architectural mechanisms, without consistently producing large errors or systematic biases that would be penalized differently by each loss function.

The following analysis focus on two key points, data characteristics and model suitability.

From the perspective of dynamic characteristics of time series, the research data exhibits typical non-stationary time series features. The time series exhibits significant trend and autocorrelation characteristics (ADF test, *p* < 0.05), indicating a gradual declining trend in mortality rates over time. This observed pattern reflects the characteristic progression of HHD, influenced by both the expanded use of antihypertensive medications (contributing to a general decline) and concurrent lifestyle changes (introducing both long-term trends and short-term fluctuations).

The performance hierarchy of the models can be directly attributed to their inherent capabilities in capturing these specific data characteristics. The inferior performance of the ARIMA model stems from its linearity and reliance on stationary assumptions. While it can capture the deterministic trend, it fails to model the complex nonlinear interactions and adapt to the non-stationary fluctuations present in the data, such as those caused by shifting lifestyle factors. In contrast, the superior and statistically equivalent performance of both LSTM and Prophet models arises from their respective abilities to handle the dataset’s nonlinearity and non-stationarity, albeit through fundamentally different mechanisms. The LSTM network excels through its dynamic gated mechanisms (e.g., forget and input gates), which allow it to autonomously learn and adapt to both the long-term declining trend and the short-term fluctuations by maintaining a cell state that propagates critical information over long time intervals. The Prophet model demonstrates its strength via its decomposable additive framework, which is inherently designed for non-stationary time series. It explicitly separates and models the long-term trend and potential seasonality components through highly interpretable parameters, making it robust against the trends and fluctuations observed in this 32-year mortality series.

Thus, the superior performance of LSTM and Prophet is not coincidental but is a direct result of their architectural compatibility with the core characteristics of the mortality data. This successful modeling of complex temporal patterns further demonstrates the validity of univariate approaches for this type of analysis.

From the model suitability perspective, each model demonstrates distinct strengths aligned with specific analytical priorities. While all four models support univariate forecasting, their architectural differences lead to varied trade-offs between predictive power, interpretability, and computational efficiency. The LSTM network provides theoretically superior capability for capturing complex nonlinear dynamics and long-term dependencies through its gated memory mechanisms, which is particularly valuable for modeling intricate temporal patterns. In contrast, Prophet offers exceptional interpretability through its decomposable additive framework that explicitly models trend, seasonality, and changepoints via intuitive parameters—this makes it especially suitable for applications requiring transparent insights into driving factors. Although statistically equivalent in overall performance for this specific dataset, the choice among these models ultimately depends on whether the analytical priority favors predictive sophistication (favoring LSTM) or interpretability and explanatory power (favoring Prophet).

### 4.2 Comparative analysis of modeling performance and disease burden prediction value among traditional statistical models, structured time series models, traditional machine learning, and deep learning models for univariate historical data

ARIMA is a classical linear regression model that characterizes time series through linear combinations of AR and MA components. Its core assumptions are stationarity and linear additivity of the series. However, it exhibited significant non-stationary characteristics (ADF test: *p* < 0.05) in the study. Despite differencing preprocessing and adequate model fitting, these inherent data properties resulted in elevated prediction errors for the ARIMA model on the validation set (MSE = 0.03645; MAE = 0.18667; MAPE = 14.162%). The DM test confirmed that these errors were statistically significantly higher than those of all other models (*p* < 0.05 for all comparisons under MSE, MAE, and MAPE loss), solidifying ARIMA as the least suitable approach for this data. Specifically, ARIMA’s linear trend assumption fails to capture the gradual deceleration in mortality decline rates. Setting the maximum autoregressive lag order at q = 1 causes the model to only capture short-term historical information, failing to account for long-term cyclical patterns. In contrast, both LSTM and Prophet overcome these limitations through their inherent architectural advantages. The LSTM model effectively captures nonlinear trends in time series by introducing gating mechanisms (e.g., input gate, forget gate, and output gate) to regulate information flow (21). The LSTM model addresses the long-term dependency problem in traditional RNNs by utilizing its internal “memory cells” and “gating mechanisms” to retain information across extended time sequences (27). Similarly, Prophet’s additive decomposition framework explicitly models non-stationary trends and seasonality through interpretable parameters. Both approaches demonstrated statistically equivalent superiority over ARIMA across all error metrics, solidifying them as superior choices for this forecasting task.

Prophet decomposes time series into trend, seasonality, and holiday effects using an additive framework. Its design emphasizes extrapolating dominant historical trends—here, the clear upward trajectory from 2011 to 2021—leading to a projected mortality increase from 2022 to 2030 (Figure 7). In contrast, LSTM, as a data-driven model, learns complex patterns across the entire series without predefined structural assumptions. This difference in approach explains the divergence: Prophet extends the recent trend, while LSTM may emphasize non-trend patterns or cyclical dynamics, resulting in a decreasing forecast. Despite these divergent projections, the DM test indicated that LSTM’s performance advantage over Prophet was not statistically significant (*p* > 0.05 for MSE, MAE, and MAPE) for this dataset. Thus, while Prophet’s forecast is a direct consequence of its design—extending dominant historical trends—LSTM’s prediction reflects its capacity to autonomously learn temporal dependencies without being bound to preset structural forms.

XGBoost is a classical machine learning model based on gradient-boosted trees. It employs lagged features for time series modeling, with its performance heavily dependent on the appropriateness of feature engineering. This study employs a time window of 4 as input. While this configuration demonstrates a statistically significant improvement over ARIMA (DM test, *p* < 0.05 for all metrics), its performance relative to Prophet depends on the error metric. Based on MAE loss, Prophet was statistically superior to XGBoost (*p* = 0.0341); similarly, under MAPE loss, Prophet also showed a significant advantage (*p* = 0.0339); however, under MSE loss, their difference was not statistically significant (*p* = 0.0762). The predictive accuracy of our model is significantly influenced by feature selection. The binary splitting mechanism of decision trees struggles to capture gradual transitions in time series, resulting in poorer stability for long-term forecasts. Compared to deep learning models, XGBoost demonstrates a 78.72% higher MSE than LSTM on the validation set; however, the DM test indicated that this difference was not statistically significant (*p* = 0.5441 for MSE; *p* = 0.1946 for MAE; *p* = 0.1951 for MAPE). This finding aligns with the conclusion of Nguyen et al. (28) for time series forecasting tasks, XGBoost’s performance lies between traditional statistical models (ARIMA) and deep learning approaches (LSTM).

LSTM is a pivotal deep learning architecture that effectively captures long-term dependencies and nonlinear patterns through its gating mechanism, demonstrating superior performance in our study. Validation results demonstrate that the LSTM model (MSE = 0.00021, MAE = 0.00872, MAPE = 0.662%) achieves a 34.05% reduction in prediction error compared to the Prophet model. However, the DM test revealed that this performance advantage was not statistically significant (*p* = 0.8762 for MSE; *p* = 0.4292 for MAE; *p* = 0.4303 for MAPE). Its capability to learn temporal dynamics significantly outperforms other models, making it a top-performing model suitable for capturing the complex trends in our dataset, alongside Prophet. There is a significant difference between the prediction results of Prophet and LSTM, while the prediction results of LSTM and XGBoost align more closely with each other (Figures 7–9). This visually demonstrates how different prediction mechanisms can produce varying outcomes and provides a methodological cross-validation between the approaches.

Our findings contribute to the existing research particularly in the statistical equivalence between the complex LSTM and the structured Prophet model. This equivalence was consistently observed across three distinct loss functions (MSE, MAE, and MAPE), each penalizing different types of forecast errors, which underscores the robustness of their performance. Previous studies in infectious disease forecasting (13, 14) have conclusively demonstrated the superiority of nonlinear models over traditional ARIMA, a conclusion our research strongly supports for chronic disease burden data. However, many existing studies in economics (7, 8) and environmental science (9, 11) tend to focus on declaring a single “best” model or on building complex ensembles. This study advances this discourse by demonstrating that for foundational forecasting tasks, statistical equivalence between top-performing models is a likely outcome. This shifts the practical question from “which model is the best?” to “which model is the most appropriate for a specific decision-making context?”

Through a rigorous comparative evaluation, this study establishes a practical framework for univariate disease burden forecasting using GBD data—an area lacking systematic methodological research. Our findings demonstrate that both LSTM and Prophet significantly outperform traditional statistical methods and are statistically equivalent for this task, a conclusion that holds true regardless of whether absolute errors (MSE, MAE) or relative percentage errors (MAPE) are considered. This provides crucial evidence-based guidance for real-world applications: in resource-limited settings requiring interpretability, Prophet is an excellent choice; for capturing highly complex temporal patterns without preset assumptions, LSTM is preferable. By employing a controlled univariate design, we ensure that the observed performance differences are attributable to the models’ inherent capabilities rather than exogenous variables, thereby offering a reliable benchmark for future research.

Limitations. While four representative models were chosen for univariate comparisons, the relatively narrow range of modeling approaches included, which is common in this research field, may constrain the generalizability of the findings. The comprehensive assessment of how univariate models perform in predicting the burden of disease still needs to be improved, due to the exclusion of other relevant models. To address this issue and enhance the generalizability of the conclusions, follow-up studies should incorporate longer time-series data, thereby further improving the reliability and application of the research findings.

## 5 Conclusion

This study conclusively demonstrated that modern forecasting models (Prophet, XGBoost, LSTM) significantly outperform traditional statistical methods (ARIMA) for predicting HHD mortality in WCBA. The LSTM and Prophet models form a top tier of statistically equivalent performance, confirmed by the Diebold-Mariano test. Prophet is ideal for interpretability and communication to stakeholders, while LSTM shows better performance in capturing the most complex, non-linear patterns. These findings offer an evidence-based framework to improve the forecasting tools of disease burden in public health, which enable the decision-makers to select the most suitable model based on the practical needs, thereby supporting better global cardiovascular disease resource allocation. Future studies ought to include longer time-series data to enhance the generalizability of conclusions.

## Data Availability

The datasets analyzed for this study can be found in the Global Burden of Disease (GBD) repository, available through the Institute for Health Metrics and Evaluation (IHME) at: http://ghdx.healthdata.org/gbd-results-tool. These are publicly available datasets, and no special access permissions are required.

## Footnotes

^1^ https://vizhub.healthdata.org/gbd-results/
